# Mortality Trends Associated With Acute Myocardial Infarction and Psychoactive Substance Use in Older Adults: A US Nationwide Analysis (1999–2020)

**DOI:** 10.1002/clc.70191

**Published:** 2025-08-11

**Authors:** Muhammad Hamza Shuja, Ramish Hannat, Ahmad Shahid, Komail Khalid Meer, Ayesha Mubbashir, Maliha Edhi, Irfan Ullah, Ahmad Alareed, Nitish Behary Paray, Raheel Ahmed, Bernardo Cortese, Michael E. Hall

**Affiliations:** ^1^ Dow University of Health Sciences Karachi Pakistan; ^2^ Services Institute of Medical Sciences (SIMS) Lahore Pakistan; ^3^ University Hospitals Cleveland Medical Center Cleveland Ohio USA; ^4^ University Hospital Southampton NHS Foundation Trust Southampton UK; ^5^ Somerset NHS Foundation Trust Taunton UK; ^6^ National Heart and Lung Institute Imperial College London London UK; ^7^ Fondazione Ricerca e Innovazione Cardiovascolare Milano Italy; ^8^ Department of Medicine University of Mississippi Medical Center Jackson Mississippi USA

**Keywords:** acute myocardial infarction, demographic disparities, mortality trends, older adults, psychoactive substance use, public health interventions

## Abstract

**Background:**

Acute myocardial infarction (AMI) remains a leading cause of mortality in the USA, particularly among individuals aged 65 and older. There is limited research about the association between psychoactive substance use and cardiovascular death due to AMI. This study aims to analyze trends in AMI‐related mortality among older adults (aged ≥ 65) associated with psychoactive substance use in the USA from 1999 to 2020, with a focus on demographic and geographic variations.

**Methods:**

We conducted a descriptive analysis using death certificates from the CDC's WONDER database. Data were extracted for age, sex, race/ethnicity, urban–rural status, and geographic region. Crude mortality rates and AAMR were calculated, and temporal trends were assessed using Joinpoint regression.

**Results:**

Between 1999 and 2020, there were 231 359 AMI‐related deaths among older adults with substance use disorders. Men (39.2) had a markedly higher mortality rate than women (15.0). Mortality rates increased across all age groups, with the most pronounced rise in those aged 85 and older (33.9). Metropolitan areas (22.3) experienced lower mortality rates than nonmetropolitan areas (37.9). The Midwest (32.3) consistently recorded the highest mortality rates, followed by the Northeast (25.0), South (24.5), and West (18.7).

**Conclusion:**

The study reveals notable temporal trends in AMI mortality among older adults with psychoactive substance use, highlighting significant demographic and regional disparities. These findings underscore the need for targeted interventions to address this growing public health issue.

## Graphical abstract

Acute myocardial infarction (AMI) mortality among older adults with psychoactive substance use has risen significantly from 1999 to 2020, with notable disparities by sex, age, race, and urban–rural status. Higher mortality rates were observed in men, nonmetropolitan areas, and non‐Hispanic Whites, highlighting the need for targeted public health interventions.

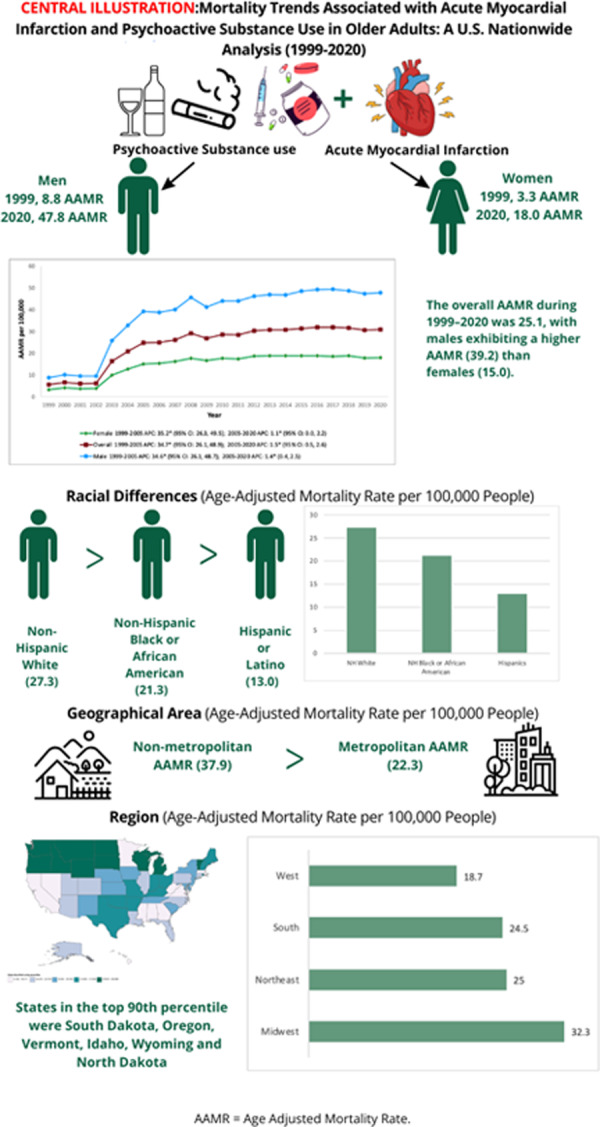

## 1 Introduction

Acute myocardial infarction (AMI) occurs when blood flow to a part of the heart is significantly reduced or blocked, leading to injury and eventual death of heart muscle tissue. This blockage is typically caused by a buildup of atherosclerotic plaque or, in some cases, by coronary artery spasm [1]. In the United States, cardiovascular disease (CVD) remains the leading cause of mortality, with its age‐adjusted death rate roughly double that of cancer [2]. AMI alone accounts for over half of these cardiovascular‐related deaths, particularly affecting people aged 65 and older [3]. The heightened risk in this older population is largely due to age‐associated physiological changes and a higher prevalence of contributing conditions such as diabetes, hypertension, dyslipidemia, and obesity.

Beyond traditional risk factors, psychoactive substance use, including marijuana, nicotine, alcohol, and opioids, has emerged as a growing public health concern among older adults. While substance use generally declines after early adulthood, nearly 1 million US adults aged 65 and older live with a substance use disorder (SUD) [4]. This is often associated with polypharmacy and unregulated medication use; studies show that over 80% of older adults take at least one potentially addictive prescription daily, with nearly half consuming five or more medications or supplements [5]. Some people get these medications through prescriptions, while others use illicit narcotics to deal with concerns including retirement, sorrow, and social isolation.

Psychoactive medications are linked to AMI through mechanisms such as thrombogenesis, endothelial dysfunction, coronary vasospasm, and elevated myocardial oxygen demand. Additionally, literature suggests that substance use raises the risk of death by exacerbating ischemic injury, producing fatal arrhythmias, and complicating medical treatment. Tobacco accelerates endothelial failure and thrombosis, opioids exacerbate hypoxia and bradyarrhythmias, and stimulants like cocaine and amphetamines cause severe vasospasm and arrhythmias [6, 7]. Cannabis disrupts autonomic regulation, whereas MDMA and hallucinogens are linked to electrolyte abnormalities and malignant arrhythmias [8]. Alcohol abuse causes coagulopathy and cardiomyopathy, which worsen the effects of AMI [9]. People with substance use disorders may delay seeking help, exhibit poor treatment adherence, and have adverse drug interactions, all of which increase mortality risks.

Despite the growing awareness of cardiovascular health risks associated with the use of psychoactive substances, very little research has been done that particularly explores its complications in patients above 65 years with AMI. Most studies either focus on the younger population or fail to address the combined impact of substance use and AMI in older individuals.

This study analyzes mortality rates among older adults with AMI who used psychoactive substances in the USA, identifying disparities based on age group, gender, race/ethnicity, urban/rural status, location, and state. The study's identification of critical demographic and regional characteristics may help future public health programs aimed at lowering cardiovascular risk and implementing drug use therapy in older adults.

## 2 Methodology

### 2.1 Study Setting and Population

We conducted a retrospective observational study by analyzing death certificates from the Centers for Disease Control and Prevention's WONDER (Wide‐Ranging Online Data for Epidemiologic Research) database, an extensive national platform in the USA that provides access to vital statistics, including mortality records across various geographic regions. This database was utilized to examine AMI mortality linked to psychoactive substance use in older adults aged 65 and above from 1999 to 2020 across the USA [10]. The data analysis relied on the International Statistical Classification of Diseases and Related Health Problems‐10th Revision (ICD‐10) codes: I21.0, I21.1, I21.2, I21.3, I21.4, and I21.9 to identify deaths associated with AMI. Notably, we did not use I22 as the study focus was to capture initial AMI episodes. The psychoactive substance use was determined using codes F10–F19, which represent substances such as alcohol, opioids, cannabis, sedatives, stimulants, and hallucinogens. Previous research has utilized these ICD‐10 codes to detect AMI and substance use disorders in administrative data sets [11, 12, 13]. The Multiple Cause of Death Public Use data set was employed to investigate instances where mortality from AMI was related to psychoactive substance use, whether as a primary cause or a contributing factor. This study was conducted using deidentified public use data provided by the government and complied with STROBE guidelines for reporting observational studies; thus, it did not need approval from a local institutional review board [14].

### 2.2 Data Abstraction and Outcomes

The data collection encompassed a range of demographic variables, including age, gender, race/ethnicity, urban–rural classification, state, and census region. Race or ethnicity was divided into categories: Hispanic (Latino), non‐Hispanic (NH) White, NH Black/African American, NH American Indian/Alaskan Native, and NH Asian [15]. The Urban–Rural Classification Scheme from the National Center for Health Statistics was employed to distinguish the population as urban (large metropolitan areas with a population of 1 million or more, and medium/small metropolitan areas with a population of 50 000 or fewer) or rural (population < 50 000). Census regions were defined as Northeast, Midwest, South, and West, following the classifications specified by the US Census Bureau [16].

### 2.3 Statistical Analysis

Crude mortality rates (CMRs) and age‑adjusted mortality rates (AAMRs) per 100,000 individuals, with 95% confidence intervals (CIs), were calculated. AAMRs were age‑standardized to the 2000 U.S. population using the direct method to evaluate trends in AMI mortality linked to psychoactive substance use from 1999 to 2020 across various demographics [17]. The CMR was calculated by dividing the total number of AMI deaths attributed to psychoactive substance use by the corresponding population in each year. The Joinpoint Regression Program (Version 5.0.2, National Cancer Institute) was utilized to examine the trends in mortality rates over time, which involved fitting log‐linear regression models to the crude data trends and computing the APC in AAMR, along with their 95% CI [17, 18]. This methodology allows for the identification of significant trends in AAMR over time by applying log‐linear regression models to capture temporal fluctuations. APCs were categorized as increasing or decreasing when the slope of the regression line significantly differed from zero, based on two‐tailed *t*‐tests. A *p *< 0.05 was considered statistically significant.

## 3 Results

Between 1999 and 2020, a total of 231 359 AMI‐related deaths were recorded among older adults with psychoactive SUDs in the United States. Men constituted 65.5% of these fatalities, and women accounted for the remaining 34.5%. Among the age brackets, 104 237 deaths occurred in individuals aged between 65 and 74, with 86 584 deaths in the 75–84‐year cohort, and 40 538 deaths in the 85+ year cohort. NH White people dominated the demographic landscape, accounting for 87.4% of these deaths, followed by NH Black or African American people (7.3%), and Hispanic or Latino people (3.5%) (Table S1). Urban counties, including large and medium/small metropolitan areas, hosted 72.7% of the total AMI and psychoactive substance use‐related deaths. Among the 231 131 individuals with documented death locations, 56.4% died in medical facilities, 30.7% at home, and 8.4% in nursing homes or long‐term care facilities (Table S2).

**Figure 1:**
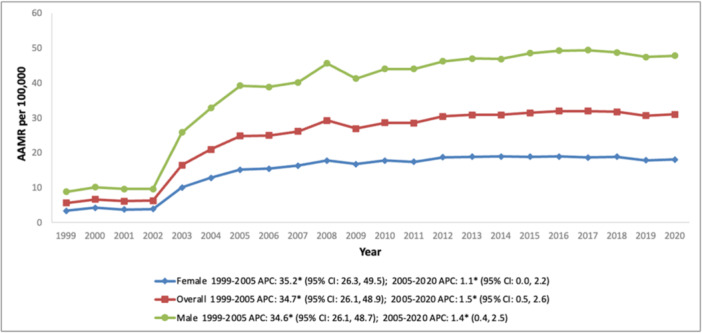
Trends in crude mortality rate for psychoactive substance‐related acute myocardial infarction in the United States (1999–2020), stratified by sex.

In the nationwide analysis, the mean AAMR for AMI‐related mortality among older adults with psychoactive SUDs stood at 25.1/100 000 individuals between 1999 and 2020. The AAMR surged from 5.6 in 1999 to 31 in 2020. The growth in AAMR was particularly pronounced between 1999 and 2005 (APC: 34.7; 95% CI: 26.1–48.9), followed by a period of gradual ascent from 2005 to 2020 (APC: 1.5; 95% CI: 0.5–2.6) (Figure 1 and Table S3).

### 3.1 Demographic Trends

#### 3.1.1 Sex

During the duration of the study, men exhibited markedly higher AAMR than women (overall AAMR men: 39.2; 95% CI: 39.0–39.4, women: 15.0; 95% CI: 14.9–15.1). The AAMR for men surged from 8.8 in 1999 to 47.8 in 2020, with a sharp rise observed between 1999 and 2005 (APC: 34.6; 95% CI: 26.1–48.7), followed by steady inflation over the subsequent 15 years (APC: 1.4; 95% CI: 0.4–2.5). The AAMR for women mirrored this trajectory, rising from 3.3 in 1999 to 15.1 in 2005 (APC: 35.2; 95% CI: 26.3–49.5), followed by a phase of subtle growth between 2005 and 2020 (APC: 1.1; 95% CI: 0.0–2.2) (Figure 1 and Figure S1, Table S3).

#### 3.1.2 Race and Ethnicity

**Figure 2:**
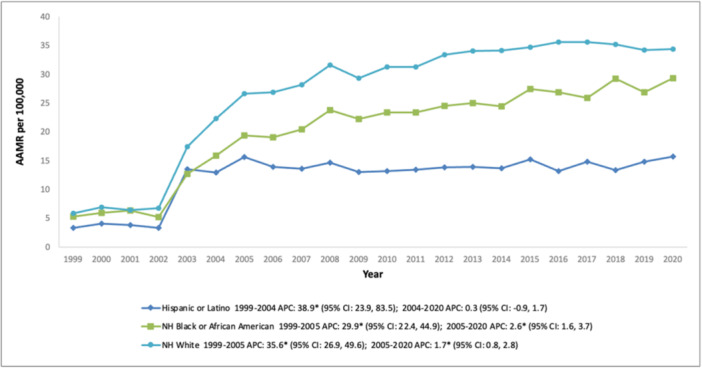
Trends in age‐adjusted mortality rate for psychoactive substance‐related acute myocardial infarction in the United States (1999–2020), stratified by race.

NH White individuals recorded the highest mean AAMR among the racial and ethnic groups, trailed by NH Black or African American individuals, and Hispanic or Latino individuals (overall AAMR NH White: 27.3; 95% CI: 27.2–27.4, NH Black or African American: 21.3; 95% CI: 21.0–21.6, Hispanic or Latino: 13.0; 95% CI: 12.7–13.3). For Hispanic or Latino individuals, the AAMR increased strikingly between 1999 and 2004 (APC: 38.9; 95% CI: 23.9–83.5), before relatively stabilizing for the remainder of the study period (APC: 0.3; 95% CI: −0.9 to 1.7). NH White and NH Black individuals displayed a comparable trajectory, characterized by a pronounced surge in AAMR from 1999 to 2005, followed by a gradual rise over the subsequent one‐and‐a‐half decades (Figure 2 and Table S4).

#### 3.1.3 Age

**Figure 3:**
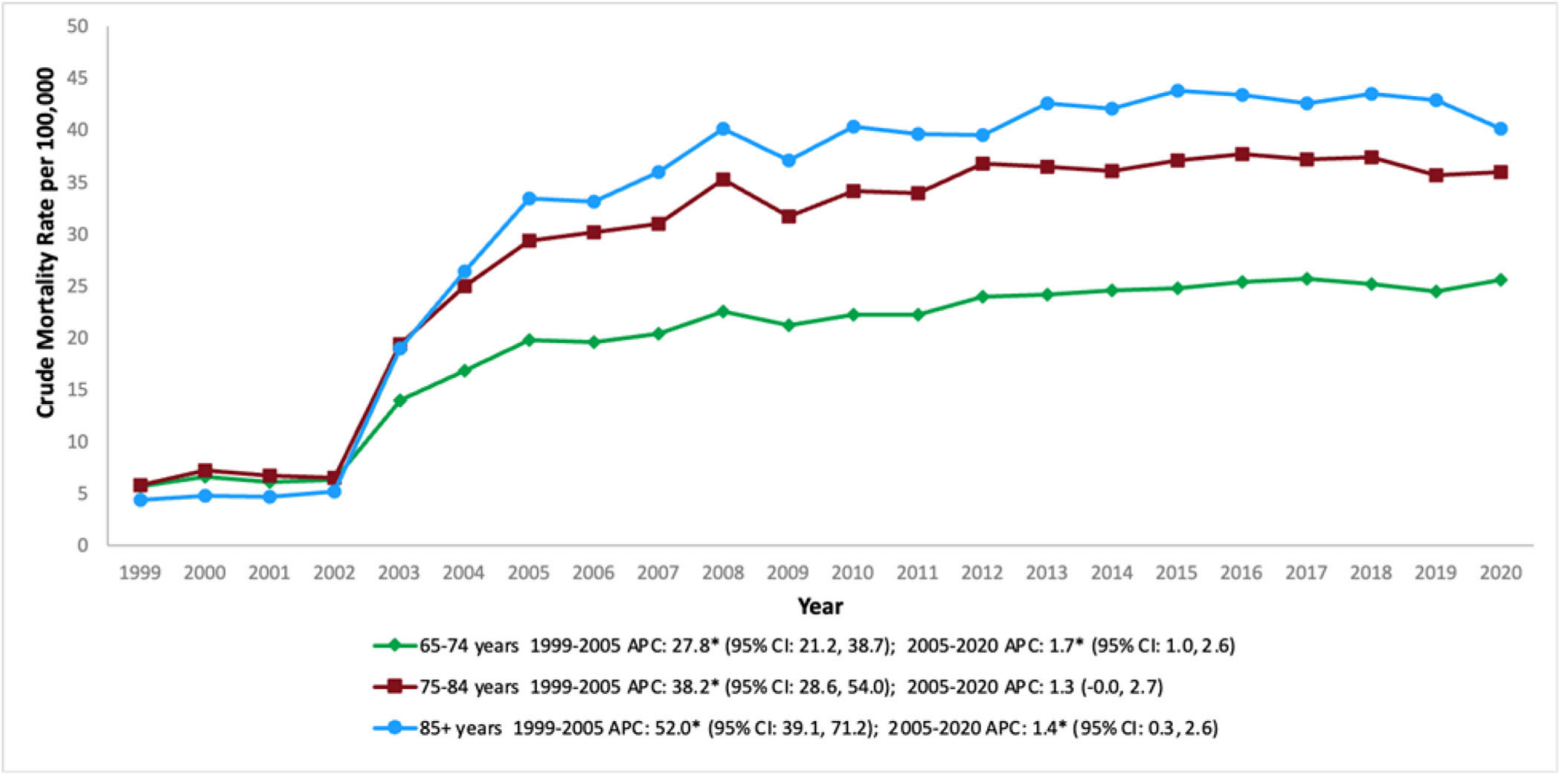
Trends in age‐adjusted mortality rate for psychoactive substance‐related acute myocardial infarction in the United States (1999–2020), stratified by age.

The CMR increased progressively with age among the studied population. Specifically, individuals aged 85 years and older experienced the highest mortality rate at 33.9 (95% CI: 33.6–34.2). Those aged 75–84 years had a CMR of 29.0 (95% CI: 28.8–29.2), while the 65–74‐year age group had the lowest rate at 20.4 (95% CI: 20.3–20.5). Each age group experienced a dramatic surge in CMR between 1999 and 2005, with the 85+ cohort witnessing the steepest climb (APC: 52.0; 95% CI: 39.1–71.2), followed by the 75–84‐year group (APC: 38.2; 95% CI: 28.6–54.0), and the 65–74‐year group (APC: 27.8; 95% CI: 21.2–38.7). From 2005 to 2020, the CMR continued to ascend steadily across all three age brackets, reflecting a persistent upward trajectory (Table 1 and Figure 3).

**Table 1:** Mortality rates per 100 000 population (with 95% confidence intervals) for acute myocardial infarction (AMI) and psychoactive substance use among adults aged ≥ 65 years in the USA from 1999 to 2020.

Demographics	Deaths	Population	Rate per 100 000 (95% CI)
Overall			
Psychoactive substance use	3 448 754	928 476 665	375.4 (375.0–375.8)
Acute myocardial infarction	3 012 647	928 476 665	326.9 (326.6–327.3)
Psychoactive substance use + acute myocardial infarction	231 359	928 476 665	25.1 (25.0–25.2)
Sex			
Men	151 550	400 844 539	39.2 (39.0–39.4)
Women	79 809	527 632 126	15.0 (14.9–15.1)
Race			
Hispanics	8094	65 124 722	13.0 (12.7–13.3)
NH White	202 126	742 709 179	27.3 (27.2–27.4)
NH Black/African American	16 902	80 673 683	21.3 (21.0‐21.6)
Metropolitan–nonmetropolitan			
Metropolitan areas	168 193	760 496 648	22.3 (22.2–22.4)
Nonmetropolitan areas	63 166	167 978 547	37.9 (37.6–38.2)
Census region			
Northeast	45 088	179 714 300	25.0 (24.8–25.2)
Midwest	66 645	207 118 111	32.3 (32.0–32.5)
South	83 063	343 442 253	24.5 (24.3–24.6)
West	36 563	198 202 001	18.7 (18.5–18.9)
Age group			
65–74	104 237	510 458 341	20.4 (20.3–20.5)^a^
75–84	86 584	298 504 433	29.0 (28.8–29.2)^a^
85+	40 538	119 513 891	33.9 (33.6–34.2)^a^

*Note:* Rates are stratified by sex, race/ethnicity, census region, and age group. Crude rates are reported for age groups.

^a^ Reported as crude rate.

### 3.2 Regional Trends

#### 3.2.1 Metropolitan–Nonmetropolitan Status

**Figure 4:**
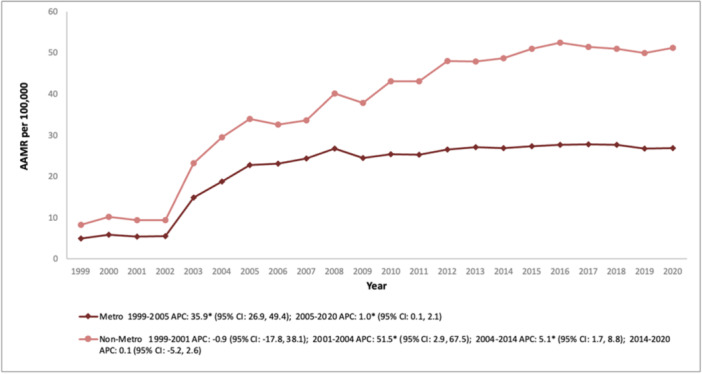
Trends in age‐adjusted mortality rate for psychoactive substance‐related acute myocardial infarction in the United States (1999–2020), stratified by metropolitan–nonmetropolitan status.

Metropolitan counties, including large and medium/small urban areas, consistently displayed lower AAMR than nonmetropolitan counties throughout the study period (overall AAMR metropolitan: 22.3; 95% CI: 22.2–22.4, nonmetropolitan: 37.9; 95% CI: 37.6–38.2). In metropolitan counties, the AAMR soared dramatically from 4.9 in 1999 to 22.7 in 2005 (APC: 35.9; 95% CI: 26.9–49.4), followed by an imperceptible rise for the remainder of the study period (APC: 1.0; 95% CI: 0.1–2.1). Nonmetropolitan counties experienced a prodigious surge, with the AAMR leaping from 8.3 in 1999 to 51.1 in 2020. The growth was marked by a drastic spike between 2001 and 2004 (APC: 51.5; 95% CI: 2.9–67.5), a gentler ascent from 2004 to 2014 (APC: 5.1; 95% CI: 1.7–8.8), and eventual stabilization during the latter years of the study (Figure 4 and Table S5).

#### 3.2.2 Geographic Region

**Figure 5:**
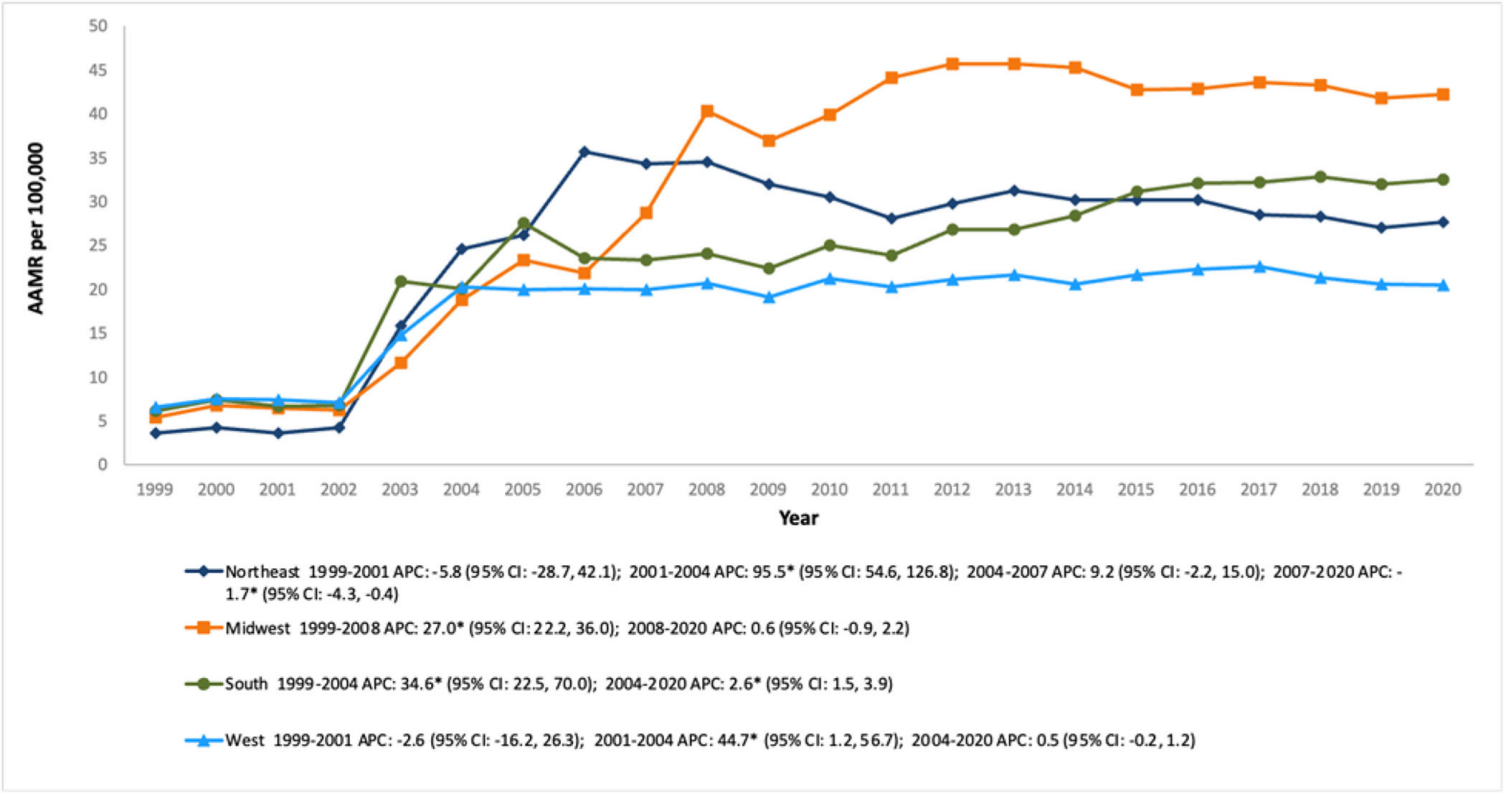
Trends in age‐adjusted mortality rate for psychoactive substance‐related acute myocardial infarction in the United States (1999–2020), stratified by census region.

The Midwest invariably recorded the highest AAMR among the four census regions, followed sequentially by the Northeast, the South, and the West (overall AAMR Midwest: 32.3; 95% CI: 32.0–32.5, Northeast: 25.0; 95% CI: 24.8–25.2, South: 24.5; 95% CI: 24.3–24.6, West: 18.7; 95% CI: 18.5–18.9). In the Northeastern region, the AAMR showed a spike between 2001 and 2004 (APC: 95.5; 95% CI: 54.6–126.8), then stabilized between 2004 to 2007 before gradually declining over the subsequent 14 years (APC: −1.7; 95% CI: −4.3 to −0.4). By contrast, the remaining three regions demonstrated a consistent upward trend in AAMR, except for a brief dip in the Western region from 1999 to 2001 (APC: −2.6; 95% CI: −16.2 to 26.3) (Figure 5 and Table S6).

#### 3.2.3 State

In the statewide analysis, the AAMR ranged from 69.1 in North Dakota to 5.1 in California. The states falling within the upper 90th percentile of AMI and psychoactive substance use‐related mortality included North Dakota, Wyoming, Idaho, Vermont, Oregon, and South Dakota. The states in the lower 10th percentile included California, Alabama, Massachusetts, Mississippi, the District of Columbia, and Virginia (Figure S2 and Table S7).

## 4 Discussion

This analysis of AMI mortality associated with psychoactive substance use in individuals over 65 reveals distinct trends across two key periods: 1999–2005 and 2005–2020. Between 1999 and 2005, the average APC in AMI mortality increased sharply at a rate of 34.7% (95% CI: 26.1–48.9), with AAMRs rising from 5.6/100 000 in 1999 to 24.8 in 2005. This initial period reflects a substantial increase in mortality; a trend likely driven by the rising prevalence of substance use, particularly prescription opioids during the early 2000s, often intended for chronic pain management in older populations [19]. It contributed significantly to increased AMI risk, as opioids can depress respiratory and cardiovascular functions, exacerbating preexisting heart conditions [20]. Additionally, high rates of nicotine and alcohol dependence further compounded cardiovascular risks for this age group, increasing susceptibility to fatal AMI events as the cumulative effects of these substances, alongside other psychoactive agents, took a toll on aging bodies [21, 22, 23, 24, 25, 26].

Minhas and colleagues examined trends in substance use and CVD‐related deaths from 1999 to 2019, finding notable increases, especially among women, young people, and residents of nonmetropolitan areas, with alcohol playing a major role [27]. However, their study did not specifically look at AMI‐related deaths in older adults, nor did it focus on tobacco use. In contrast, our study found that tobacco use accounted for the majority of deaths among older adults, highlighting its ongoing impact in this group. It is important to note that older adults are more vulnerable due to age‐related changes and underrepresentation in research. Our study reveals significant regional and demographic differences, with higher death rates in rural communities and among people aged 85 and older. These results highlight the pressing need for targeted public health strategies to tackle the unique challenges faced by this vulnerable group.

From 2005 to 2020, however, the rate of increase in AMI‐related mortality decelerated, with an APC of 1.5% (95% CI: 0.5–2.6). This shift is attributed to the impact of significant advancements in cardiovascular care, especially in the management and prevention of AMI, such as improved reperfusion therapies, developments in stent procedures, and the introduction and widespread use of better anticoagulant agents [28]. Such progress reduced the risk of undesirable outcomes in individuals experiencing AMI, especially among those with heightened vulnerability due to psychoactive substance use. In addition, public health campaigns aimed at reducing smoking and addressing substance abuse among older adults influenced the trend positively; For instance, smoking cessation programs and campaigns, particularly those targeting older adults, may have mitigated the cardiovascular damage caused by nicotine, thereby contributing to reductions in AMI risk among nicotine‐dependent individuals [29]. Public policies such as establishing stricter regulations on opioid prescribing in response to the opioid crisis, helped reduce the associated cardiovascular risks in the aging population [30].

Moreover, these observed trends in AMI mortality reflect multifaceted, complex health challenges faced by aging populations. Older individuals commonly manage multiple comorbidities, including hypertension, diabetes, and kidney disease, all of which significantly increase vulnerability and susceptibility to the cardiovascular effects of psychoactive substances [31]. Furthermore, polypharmacy as well as unregulated medication intake in older adults can amplify the cardiovascular risks of substance use due to potential drug interactions [32].

When comparing gender‐based trends, significant differences were observed, especially the higher mortality rates among males. Biologically, males are more susceptible to CVDs, and the impact of psychoactive substances like alcohol, nicotine, and stimulants tends to be more conspicuous in males due to said differences in cardiovascular physiology. Additionally, males generally engage in more intense substance use and riskier behaviors [33], such as excessive drinking and smoking, significantly elevating the risk of AMI. In contrast, females tend to exhibit more controlled patterns of substance use, which may contribute to their relatively stable mortality rates. Furthermore, females are more inclined to seek healthcare services [34]; early diagnosis leads to a good prognosis.

In terms of race, these trends displayed notable disparities among African Americans, Whites, and Latinos, driven by variations in healthcare availability, socioeconomic factors, and systemic inequities. African Americans experienced a significant increase in mortality rates (1999–2005, APC 29.9%), followed by a slower rise (2005–2020, APC 2.6%), with AAMRs fluctuating frequently. These patterns likely stem from a greater burden of comorbidities, barriers to accessing medical care, and systemic challenges that heighten the cardiovascular impact of psychoactive substance use. White individuals saw a sharper escalation in early years (1999–2005, APC 35.6%), stabilizing slightly afterward (2005–2020, APC 1.7%), with consistently higher AAMRs than other racial groups. This trajectory may be influenced by the disproportionate impact of the opioid epidemic on White populations and differences in preventive care outreach [35]. Latinos showed an initial steep rise (1999–2004, APC 38.9%) that leveled off (2004–2020, APC 0.3%), maintaining relatively lower AAMRs, possibly due to stronger familial and community support networks.

Differences were also observed when comparing metropolitan and nonmetropolitan areas. Metropolitan areas experienced a sharp rise in mortality rates from 1999 to 2005, followed by a slower, steady increase through 2020. In contrast, nonmetropolitan areas showed erratic trends, with a slight decline from 1999 to 2001, a steep rise from 2001 to 2004, and moderate increases until 2014, stabilizing thereafter. These differences can be attributed to disparities in healthcare access, with metropolitan areas benefiting from better infrastructure, earlier diagnoses, and advanced interventions, while rural regions face delays and limited resources [36]. Socioeconomic challenges such as lower income levels and lack of education or literacy in nonmetropolitan areas further exacerbate the observed health disparities. Moreover, substance use patterns and public health initiatives vary in comparison, with metropolitan areas often benefiting from comprehensive campaigns and broader support systems [37].

Similarly, geographic patterns across the United States also reveal striking differences in AMI mortality trends, influenced by regional disparities in healthcare infrastructure, socioeconomic status, and population demographics. The Northeast experienced an initial decline (1999–2001) preceding a sharp increase (2001–2004), and a gradual decrease concluding the trends, reflecting the opioid epidemic's impact being countered by constantly improving healthcare access. The Midwest saw a significant rise in mortality (1999–2008), stabilizing after 2008. The rise is likely due to high substance use rates and rural healthcare access challenges. The South exhibited a sharp increase (1999–2004) with steady growth through 2020, reflecting systemic healthcare inequities and higher rates of comorbid conditions. In contrast, the West showed relatively stable trends, with minor fluctuations and lower mortality rates, attributed to stronger public health policies and better access to preventive care.

These intersecting disparities across geography, ethnicity, and socioeconomic status highlight the pressing need for more granular research to address the root causes of these inequities in AMI mortality.

## 5 Study Limitations

Several limitations in the study design and data sources may have influenced the findings. One major challenge lies in accurately identifying AMI as the underlying cause of death. Diagnostic errors and variations in death certificate reporting can sometimes result in misclassification of the disease, compromising the reliability of AMI‐related mortality data [38]. Additionally, mortality data specific to NH‐American Indian/Alaska Natives and NH‐Asian‐American populations were considered unreliable in the CDC WONDER database, preventing a detailed subgroup analysis for these communities.

Another limitation arises from potential inconsistencies in how health records were documented and reported across different regions; this might have introduced regional bias. Adding to that, our study also lacked access to critical clinical information such as cardiovascular risk profiles, HIV status, and the specific types of psychoactive substances involved (e.g., alcohol, opioids, cannabis, etc.). These missing data points restricted our ability to investigate the nuanced influencers of AMI mortality and how these may have changed over time.

Moreover, our analysis was unable to assess the temporal relationship between substance use and the onset of AMI or death as an outcome. An ideal and precise approach would involve examining patients who died within a specific time frame, for example, death within 6–12 months after diagnosis of AMI; data that were not accessible. It would have allowed for a better understanding of causality. Lastly, important confounding factors such as behavioral habits, comorbid conditions, and socioeconomic status were not accounted for in this study. Future research should aim to incorporate these variables to offer a more comprehensive picture of AMI‐related mortality and the role of substance use.

## 6 Conclusion

This study highlights how the influence of psychoactive substance use on AMI‐related deaths among adults over 65 has shifted over time. Between 1999 and 2005, mortality rates climbed steeply, largely due to the rise of the opioid crisis and increasing substance use. Since then, the pace of increase has slowed, likely due to improvements in both clinical interventions and public health efforts. Still, not all groups have benefited equally. Men, NH‐White individuals, and people living in rural areas continue to face disproportionately high mortality rates. These differences seemingly stem from unequal access to healthcare, socioeconomic challenges, and deeper systemic issues. There remains a pressing need for more focused research and policy efforts to understand and close these gaps, especially for the communities most at risk.

## Conflicts of Interest

The authors declare no conflicts of interest.

## Supporting information


**Supporting Figure 1:** Annual percent change in AMI mortality among older adults with psychoactive Substance Use, by Sex (1999–2020). **Supporting Figure 2:** Trends in Age‐Adjusted Mortality Rates for Psychoactive Substance‐Related Acute Myocardial Infarction in the United States (1999–2020), Stratified by States. **Supporting Table 1:** Absolute number of psychoactive substance‐related AMI deaths in adults aged 65 and above, stratified by Gender and Race in the U.S., 1999–2020. **Supporting Table 2:** Absolute number of psychoactive substance‐related AMI deaths in adults aged 65 and above, stratified by Place Of Death in the U.S., 1999–2020. **Supporting Table 3:** AAMR per 100,000 for AMI with psychoactive substance use in adults aged 65 and above, stratified by Gender in the U.S., 1999–2020. **Supporting Table 4:** AAMR per 100,000 for AMI with psychoactive substance use in adults aged 65 and above, stratified by Race in the U.S., 1999–2020. **Supporting Table 5:** AAMR per 100,000 for AMI with psychoactive substance use in adults aged 65 and above, stratified by Rural‐Urban classification in the U.S., 1999–2020. **Supporting Table 6:** AAMR per 100,000 for AMI with psychoactive substance use in adults aged 65 and above, stratified by Census Region in the U.S., 1999–2020. **Supporting Table 7:** AAMR per 100,000 for AMI with psychoactive substance use in adults aged 65 and above, stratified by State in the U.S., 1999–2020.

## Data Availability

All data generated or analyzed during this study are included in this published article and its supporting information files and are freely available on the CDC WONDER database.
