# Supplementary material for: Mortality Trends Associated With Acute Myocardial Infarction and Psychoactive Substance Use in Older Adults: A US Nationwide Analysis (1999–2020)
Source: Clin Cardiol. 2025 Aug 11;48(8):e70191. doi: 10.1002/clc.70191 (PMC12337155; doi:10.1002/clc.70191)
Supplement: Supplementary file 1 — Supporting Figure 1: Annual percent change in AMI mortality among older adults with psychoactive Substance Use, by Sex (1999–2020). Supporting Figure 2: Trends in Age‐Adjusted Mortality Rates for Psychoactive Substance‐Related Acute Myocardial Infarction in the United States (1999–2020), Stratified by States. Supporting Table 1: Absolute number of psychoactive substance‐related AMI deaths in adults aged 65 and above, stratified by Gender and Race in the U.S., 1999–2020. Supporting Table 2: Absolute number of psychoactive substance‐related AMI deaths in adults aged 65 and above, stratified by Place Of Death in the U.S., 1999–2020. Supporting Table 3: AAMR per 100,000 for AMI with psychoactive substance use in adults aged 65 and above, stratified by Gender in the U.S., 1999–2020. Supporting Table 4: AAMR per 100,000 for AMI with psychoactive substance use in adults aged 65 and above, stratified by Race in the U.S., 1999–2020. Supporting Table 5: AAMR per 100,000 for AMI with psychoactive substance use in adults aged 65 and above, stratified by Rural‐Urban classification in the U.S., 1999–2020. Supporting Table 6: AAMR per 100,000 for AMI with psychoactive substance use in adults aged 65 and above, stratified by Census Region in the U.S., 1999–2020. Supporting Table 7: AAMR per 100,000 for AMI with psychoactive substance use in adults aged 65 and above, stratified by State in the U.S., 1999–2020. [file CLC-48-e70191-s001.docx]

**Supplemental Figure 1:** Annual Percent Change in AMI Mortality Among Older Adults With Psychoactive Substance Use, by Sex (1999–2020)

**
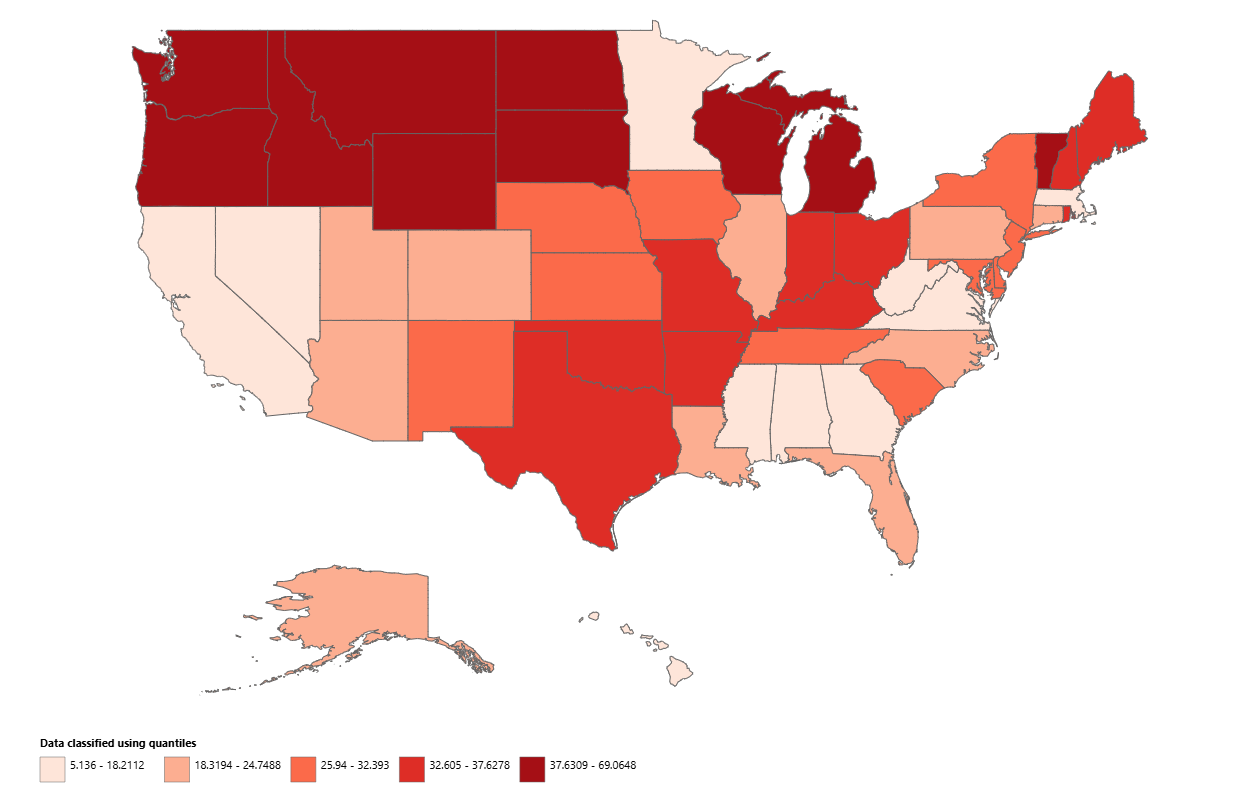
**

**Supplemental Figure 2:** Trends in Age-Adjusted Mortality Rates for Psychoactive Substance-Related Acute Myocardial Infarction in the United States (1999–2020), Stratified by States.

| **Deaths** | | | | | | | | | |
| --- | --- | --- | --- | --- | --- | --- | --- | --- | --- |
| **Year** | **Overall** | **Women** | **Men** | **NH White** | **NH Black or African American** | **NH American Indian or Alaska Native** | **NH Asian or Pacific Islander** | **Hisoanics** | **Population** |
| **1999** | 1934 | 679 | 1255 | 1692 | 148 | 21 | 13 | 53 | 34797841 |
| **2000** | 2309 | 858 | 1451 | 2032 | 169 | 19 | 12 | 68 | 34991753 |
| **2001** | 2170 | 770 | 1400 | 1887 | 182 | 15 | 11 | 68 | 35290291 |
| **2002** | 2213 | 795 | 1418 | 1963 | 153 | 13 | 13 | 65 | 35522207 |
| **2003** | 5912 | 2122 | 3790 | 5199 | 370 | 33 | 31 | 260 | 35863529 |
| **2004** | 7560 | 2727 | 4833 | 6710 | 471 | 54 | 44 | 265 | 36203319 |
| **2005** | 9120 | 3257 | 5863 | 8074 | 586 | 64 | 58 | 330 | 36649798 |
| **2006** | 9291 | 3361 | 5930 | 8240 | 586 | 71 | 69 | 309 | 37164107 |
| **2007** | 9893 | 3625 | 6268 | 8770 | 637 | 67 | 88 | 312 | 37825711 |
| **2008** | 11305 | 3996 | 7309 | 10001 | 766 | 71 | 100 | 353 | 38777621 |
| **2009** | 10628 | 3835 | 6793 | 9394 | 727 | 61 | 99 | 332 | 39623175 |
| **2010** | 11481 | 4112 | 7369 | 10159 | 778 | 71 | 101 | 352 | 40267984 |
| **2011** | 11741 | 4124 | 7617 | 10357 | 815 | 66 | 101 | 384 | 41394141 |
| **2012** | 12941 | 4548 | 8393 | 11380 | 892 | 89 | 122 | 422 | 43145356 |
| **2013** | 13554 | 4706 | 8848 | 11928 | 954 | 71 | 131 | 444 | 44704074 |
| **2014** | 13989 | 4863 | 9126 | 12272 | 969 | 94 | 149 | 470 | 46243211 |
| **2015** | 14722 | 4974 | 9748 | 12751 | 1134 | 98 | 141 | 553 | 47760852 |
| **2016** | 15381 | 5131 | 10250 | 13377 | 1173 | 122 | 156 | 512 | 49244195 |
| **2017** | 15867 | 5206 | 10661 | 13745 | 1185 | 122 | 178 | 598 | 50858679 |
| **2018** | 16256 | 5381 | 10875 | 13964 | 1381 | 127 | 181 | 559 | 52431193 |
| **2019** | 16208 | 5267 | 10941 | 13911 | 1327 | 122 | 159 | 653 | 54058263 |
| **2020** | 16884 | 5472 | 11412 | 14320 | 1499 | 125 | 165 | 732 | 55659365 |
| **Total** | 231359 | 79809 | 151550 | 202126 | 16902 | 1596 | 2122 | 8094 | 9.28E+08 |

**Supplementary Table 1:** Absolute number of psychoactive substance-related AMI deaths in adults aged 65 and above, stratified by Gender and Race in the U.S., 1999-2020.

|  | **Deaths** | | | |  |  |
| --- | --- | --- | --- | --- | --- | --- |
| **Year** | **Medical Facility** | **Home** | **Hospice** | **Nursing home/long term care** | **Other Places** | **Unknown** |
| 1999 | 1227 | 516 | missing | 132 | 58 | missing |
| 2000 | 1416 | 664 | missing | 177 | 52 | missing |
| 2001 | 1325 | 606 | missing | 168 | 69 | missing |
| 2002 | 1297 | 693 | missing | 164 | 58 | missing |
| 2003 | 3714 | 1454 | missing | 577 | 153 | 10 |
| 2004 | 4728 | 1865 | missing | 773 | 168 | 19 |
| 2005 | 5760 | 2164 | 54 | 907 | 220 | 15 |
| 2006 | 5703 | 2354 | 98 | 889 | 211 | 36 |
| 2007 | 6069 | 2502 | 107 | 974 | 214 | 27 |
| 2008 | 6946 | 2853 | 178 | 1066 | 235 | 27 |
| 2009 | 6342 | 2860 | 185 | 955 | 254 | 32 |
| 2010 | 6900 | 3094 | 190 | 1046 | 244 | missing |
| 2011 | 6919 | 3237 | 215 | 1071 | 296 | missing |
| 2012 | 7460 | 3673 | 248 | 1188 | 364 | missing |
| 2013 | 7864 | 3886 | 302 | 1182 | 316 | missing |
| 2014 | 7822 | 4328 | 346 | 1191 | 292 | 10 |
| 2015 | 7995 | 4760 | 373 | 1226 | 359 | missing |
| 2016 | 8274 | 5112 | 416 | 1207 | 372 | missing |
| 2017 | 8313 | 5481 | 455 | 1204 | 407 | missing |
| 2018 | 8240 | 5899 | 485 | 1154 | 476 | missing |
| 2019 | 8112 | 6024 | 471 | 1133 | 466 | missing |
| 2020 | 7843 | 6984 | 466 | 1056 | 529 | missing |
| Total | 130269 | 71009 | 4600 | 19440 | 5813 | 228 |
|  |  |  |  |  |  |  |

**Supplementary Table 2:** Absolute number of psychoactive substance-related AMI deaths in adults aged 65 and above, stratified by Place Of Death in the U.S., 1999-2020.

| **Year** | **Overall (95% CI)** | **Men (95% CI)** | **Women (95% CI)** |
| --- | --- | --- | --- |
| 1999 | 5.6 (5.3–5.8) | 8.8 (8.3–9.3) | 3.3 (3.1–3.6) |
| 2000 | 6.6 (6.3–6.9) | 10.1 (9.5–10.6) | 4.2 (3.9–4.5) |
| 2001 | 6.1 (5.9–6.4) | 9.6 (9.1–10.1) | 3.7 (3.5–4.0) |
| 2002 | 6.2 (6.0–6.5) | 9.6 (9.1–10.1) | 3.8 (3.6–4.1) |
| 2003 | 16.4 (16.0–16.8) | 25.8 (25.0–26.6) | 10.0 (9.6–10.4) |
| 2004 | 20.9 (20.4–21.3) | 32.8 (31.9–33.8) | 12.8 (12.3–13.2) |
| 2005 | 24.8 (24.3–25.3) | 39.2 (38.2–40.2) | 15.1 (14.5–15.6) |
| 2006 | 24.9 (24.4–25.4) | 38.8 (37.8–39.8) | 15.4 (14.8–15.9) |
| 2007 | 26.1 (25.6–26.6) | 40.1 (39.1–41.1) | 16.3 (15.8–16.9) |
| 2008 | 29.2 (28.6–29.7) | 45.6 (44.6–46.7) | 17.7 (17.1–18.3) |
| 2009 | 26.9 (26.4–27.4) | 41.2 (40.3–42.2) | 16.7 (16.2–17.3) |
| 2010 | 28.6 (28.1–29.2) | 44.0 (43.0–45.0) | 17.7 (17.2–18.3) |
| 2011 | 28.5 (28.0–29.0) | 44.0 (43.0–45.0) | 17.4 (16.9–17.9) |
| 2012 | 30.4 (29.9–30.9) | 46.2 (45.2–47.2) | 18.7 (18.1–19.2) |
| 2013 | 30.8 (30.2–31.3) | 47.0 (46.0–48.0) | 18.8 (18.2–19.3) |
| 2014 | 30.8 (30.3–31.3) | 46.8 (45.8–47.8) | 18.9 (18.3–19.4) |
| 2015 | 31.4 (30.9–32.0) | 48.5 (47.5–49.5) | 18.8 (18.2–19.3) |
| 2016 | 31.9 (31.4–32.4) | 49.2 (48.2–50.1) | 18.9 (18.4–19.4) |
| 2017 | 31.9 (31.4–32.4) | 49.4 (48.4–50.3) | 18.6 (18.1–19.1) |
| 2018 | 31.7 (31.2–32.2) | 48.7 (47.7–49.6) | 18.8 (18.3–19.3) |
| 2019 | 30.6 (30.2–31.1) | 47.4 (46.4–48.3) | 17.8 (17.3–18.3) |
| 2020 | 31.0 (30.5–31.5) | 47.8 (46.9–48.7) | 18.0 (17.6–18.5) |
| **Overall AAMR** | **25.1 (25.0–25.2)** | **39.2 (39.0–39.4)** | **15.0 (14.9–15.1)** |
|  |  |  |  |

**Supplementary Table 3:** AAMR per 100,000 for AMI with psychoactive substance use in adults aged 65 and above, stratified by Gender in the U.S., 1999-2020

| **Year** | **Black or African American (95% CI)** | **White (95% CI)** | **Hispanics (95% CI)** |
| --- | --- | --- | --- |
| 1999 | 5.3 (4.4–6.1) | 5.8 (5.5–6.1) | 3.3 (2.5–4.3) |
| 2000 | 5.9 (5.0–6.8) | 6.9 (6.6–7.2) | 4.0 (3.1–5.1) |
| 2001 | 6.3 (5.3–7.2) | 6.4 (6.1–6.7) | 3.8 (3.0–4.9) |
| 2002 | 5.2 (4.3–6.0) | 6.7 (6.4–7.0) | 3.3 (2.6–4.3) |
| 2003 | 12.7 (11.4–14.0) | 17.4 (16.9–17.9) | 13.5 (11.8–15.2) |
| 2004 | 15.9 (14.4–17.3) | 22.3 (21.7–22.8) | 12.9 (11.3–14.5) |
| 2005 | 19.4 (17.8–21.0) | 26.6 (26.0–27.1) | 15.6 (13.9–17.3) |
| 2006 | 19.0 (17.4–20.5) | 26.9 (26.3–27.4) | 13.9 (12.3–15.4) |
| 2007 | 20.4 (18.8–22.0) | 28.2 (27.6–28.8) | 13.6 (12.1–15.1) |
| 2008 | 23.8 (22.1–25.5) | 31.6 (31.0–32.3) | 14.6 (13.1–16.1) |
| 2009 | 22.2 (20.6–23.8) | 29.3 (28.7–29.9) | 13.0 (11.6–14.4) |
| 2010 | 23.4 (21.7–25.0) | 31.3 (30.7–31.9) | 13.2 (11.8–14.6) |
| 2011 | 23.4 (21.7–25.0) | 31.3 (30.7–31.9) | 13.4 (12.1–14.8) |
| 2012 | 24.5 (22.9–26.1) | 33.4 (32.7–34.0) | 13.8 (12.5–15.1) |
| 2013 | 25.0 (23.4–26.6) | 34.0 (33.4–34.6) | 13.9 (12.6–15.2) |
| 2014 | 24.4 (22.8–25.9) | 34.1 (33.5–34.7) | 13.7 (12.5–15.0) |
| 2015 | 27.4 (25.7–29.0) | 34.7 (34.1–35.3) | 15.2 (13.9–16.5) |
| 2016 | 26.9 (25.3–28.5) | 35.6 (35.0–36.2) | 13.2 (12.1–14.4) |
| 2017 | 25.9 (24.4–27.4) | 35.6 (35.0–36.2) | 14.8 (13.6–16.0) |
| 2018 | 29.2 (27.7–30.8) | 35.2 (34.6–35.8) | 13.3 (12.2–14.4) |
| 2019 | 26.9 (25.5–28.4) | 34.2 (33.6–34.8) | 14.8 (13.6–15.9) |
| 2020 | 29.3 (27.8–30.8) | 34.4 (33.8–35.0) | 15.7 (14.5–16.8) |
| **Overall AAMR** | **21.3 (21.0–21.6)** | **27.3 (27.2–27.4)** | **13.0 (12.7–13.3)** |

**Supplementary Table 4:** AAMR per 100,000 for AMI with psychoactive substance use in adults aged 65 and above, stratified by Race in the U.S., 1999-2020.

| **Year** | **Metropolitan (95% CI)** | **Non-metropolitan (95% CI)** |
| --- | --- | --- |
| 1999 | 4.9 (4.6–5.1) | 8.3 (7.6–9.0) |
| 2000 | 5.8 (5.5–6.1) | 10.2 (9.4–10.9) |
| 2001 | 5.4 (5.1–5.7) | 9.4 (8.7–10.1) |
| 2002 | 5.5 (5.2–5.8) | 9.4 (8.6–10.1) |
| 2003 | 14.9 (14.5–15.3) | 23.2 (22.0–24.3) |
| 2004 | 18.8 (18.3–19.3) | 29.5 (28.2–30.8) |
| 2005 | 22.7 (22.2–23.2) | 33.9 (32.6–35.3) |
| 2006 | 23.1 (22.6–23.7) | 32.5 (31.2–33.9) |
| 2007 | 24.3 (23.8–24.9) | 33.6 (32.3–35.0) |
| 2008 | 26.7 (26.1–27.3) | 40.1 (38.7–41.6) |
| 2009 | 24.4 (23.9–25.0) | 37.8 (36.3–39.2) |
| 2010 | 25.4 (24.8–25.9) | 43.1 (41.6–44.6) |
| 2011 | 25.3 (24.8–25.9) | 43.1 (41.6–44.6) |
| 2012 | 26.5 (25.9–27.0) | 48.0 (46.5–49.6) |
| 2013 | 27.1 (26.5–27.6) | 47.8 (46.2–49.3) |
| 2014 | 26.9 (26.4–27.4) | 48.6 (47.1–50.1) |
| 2015 | 27.3 (26.8–27.8) | 50.9 (49.4–52.5) |
| 2016 | 27.6 (27.0–28.1) | 52.4 (50.8–53.9) |
| 2017 | 27.8 (27.3–28.3) | 51.4 (49.8–52.9) |
| 2018 | 27.7 (27.2–28.2) | 50.9 (49.4–52.4) |
| 2019 | 26.7 (26.3–27.2) | 49.9 (48.4–51.3) |
| 2020 | 26.9 (26.4–27.4) | 51.1 (49.7–52.6) |
| **Overall AAMR** | **22.3 (22.2–22.4)** | **37.9 (37.6–38.2)** |

**Supplementary Table 5:** AAMR per 100,000 for AMI with psychoactive substance use in adults aged 65 and above, stratified by Rural-Urban classification in the U.S., 1999-2020.

| **Year** | **Northeast (95% CI)** | **Midwest (95% CI)** | **South (95% CI)** | **West (95% CI)** |
| --- | --- | --- | --- | --- |
| 1999 | 3.6 (3.2–4.1) | 5.4 (4.9–5.9) | 6.2 (5.8–6.6) | 6.6 (6.0–7.2) |
| 2000 | 4.2 (3.8–4.7) | 6.8 (6.3–7.4) | 7.4 (6.9–7.9) | 7.5 (6.8–8.1) |
| 2001 | 3.6 (3.2–4.1) | 6.5 (5.9–7.0) | 6.7 (6.3–7.2) | 7.4 (6.7–8.0) |
| 2002 | 4.2 (3.7–4.7) | 6.3 (5.8–6.8) | 6.8 (6.4–7.3) | 7.1 (6.5–7.7) |
| 2003 | 15.8 (14.9–16.7) | 11.6 (10.9–12.4) | 20.9 (20.1–21.7) | 14.8 (13.9–15.6) |
| 2004 | 24.6 (23.5–25.7) | 18.8 (17.9–19.7) | 20.1 (19.4–20.9) | 20.3 (19.3–21.4) |
| 2005 | 26.2 (25.1–27.4) | 23.3 (22.2–24.3) | 27.6 (26.7–28.5) | 20.0 (19.0–21.0) |
| 2006 | 35.7 (34.3–37.0) | 21.9 (20.9–22.9) | 23.5 (22.7–24.3) | 20.1 (19.1–21.1) |
| 2007 | 34.3 (33.0–35.6) | 28.7 (27.6–29.8) | 23.3 (22.5–24.1) | 20.0 (19.0–20.9) |
| 2008 | 34.5 (33.2–35.9) | 40.3 (39.0–41.6) | 24.1 (23.3–24.9) | 20.7 (19.7–21.7) |
| 2009 | 32.0 (30.8–33.3) | 36.9 (35.6–38.1) | 22.4 (21.6–23.1) | 19.1 (18.2–20.1) |
| 2010 | 30.5 (29.3–31.7) | 39.9 (38.5–41.2) | 25.0 (24.2–25.8) | 21.2 (20.2–22.1) |
| 2011 | 28.1 (26.9–29.2) | 44.1 (42.7–45.5) | 23.9 (23.2–24.7) | 20.3 (19.4–21.2) |
| 2012 | 29.8 (28.6–31.0) | 45.7 (44.3–47.0) | 26.8 (25.9–27.6) | 21.1 (20.2–22.1) |
| 2013 | 31.3 (30.1–32.5) | 45.7 (44.4–47.1) | 26.8 (26.0–27.6) | 21.7 (20.8–22.7) |
| 2014 | 30.2 (29.0–31.4) | 45.3 (44.0–46.7) | 28.4 (27.6–29.2) | 20.6 (19.7–21.5) |
| 2015 | 30.2 (29.1–31.4) | 42.7 (41.4–44.0) | 31.1 (30.3–31.9) | 21.7 (20.8–22.7) |
| 2016 | 30.2 (29.1–31.4) | 42.9 (41.6–44.2) | 32.1 (31.2–32.9) | 22.3 (21.3–23.2) |
| 2017 | 28.5 (27.4–29.6) | 43.6 (42.4–44.9) | 32.2 (31.4–33.0) | 22.6 (21.7–23.5) |
| 2018 | 28.3 (27.2–29.3) | 43.3 (42.1–44.6) | 32.8 (32.0–33.7) | 21.3 (20.5–22.2) |
| 2019 | 27.0 (26.0–28.1) | 41.8 (40.6–43.0) | 32.0 (31.2–32.8) | 20.6 (19.8–21.5) |
| 2020 | 27.7 (26.6–28.7) | 42.2 (41.0–43.4) | 32.5 (31.7–33.3) | 20.5 (19.7–21.3) |
| **Overall AAMR** | **25.0 (24.8–25.2)** | **32.3 (32.0–32.5)** | **24.5 (24.3–24.6)** | **18.7 (18.5–18.9)** |

**Supplementary Table 6:** AAMR per 100,000 for AMI with psychoactive substance use in adults aged 65 and above, stratified by Census Region in the U.S., 1999-2020.

| **State** | **AAMR (95% CI)** | **Percentile (%)** |
| --- | --- | --- |
| California | 5.1 (5.0 - 5.3) | 0.0 |
| Alabama | 7.5 (7.0 - 7.9) | 2.0 |
| Massachusetts | 8.4 (8.0 - 8.8) | 4.0 |
| Mississippi | 10.9 (10.2 - 11.6) | 6.0 |
| District of Columbia | 12.3 (10.6 - 14.0) | 8.0 |
| Virginia | 13.3 (12.9 - 13.8) | 10.0 |
| Nevada | 13.9 (13.0 - 14.8) | 12.0 |
| West Virginia | 14.5 (13.6 - 15.4) | 14.0 |
| Georgia | 14.7 (14.2 - 15.2) | 16.0 |
| Minnesota | 17.9 (17.3 - 18.6) | 18.0 |
| Hawaii | 18.2 (17.0 - 19.5) | 20.0 |
| Connecticut | 18.3 (17.5 - 19.1) | 22.0 |
| Florida | 19.5 (19.2 - 19.9) | 24.0 |
| Arizona | 20.6 (20.0 - 21.2) | 26.0 |
| Illinois | 21.3 (20.9 - 21.8) | 28.0 |
| Utah | 21.4 (20.2 - 22.7) | 30.0 |
| Colorado | 21.5 (20.7 - 22.3) | 32.0 |
| Alaska | 22.1 (19.3 - 24.8) | 34.0 |
| North Carolina | 22.7 (22.1 - 23.2) | 36.0 |
| Louisiana | 23.2 (22.4 - 24.1) | 38.0 |
| Pennsylvania | 24.7 (24.3 - 25.2) | 40.0 |
| New York | 25.9 (25.5 - 26.3) | 42.0 |
| Delaware | 27.3 (25.4 - 29.2) | 44.0 |
| Tennessee | 27.4 (26.6 - 28.1) | 46.0 |
| Iowa | 27.5 (26.5 - 28.5) | 48.0 |
| South Carolina | 27.5 (26.7 - 28.4) | 50.0 |
| New Mexico | 28.0 (26.7 - 29.4) | 52.0 |
| Nebraska | 29.3 (27.9 - 30.7) | 54.0 |
| Kansas | 31.7 (30.5 - 32.9) | 56.0 |
| New Jersey | 31.7 (31.0 - 32.4) | 58.0 |
| Maryland | 32.4 (31.5 - 33.3) | 60.0 |
| Missouri | 32.6 (31.8 - 33.4) | 62.0 |
| Maine | 33.1 (31.5 - 34.7) | 64.0 |
| Indiana | 33.5 (32.7 - 34.4) | 66.0 |
| Arkansas | 35.0 (33.8 - 36.2) | 68.0 |
| Ohio | 35.2 (34.6 - 35.8) | 70.0 |
| Kentucky | 35.4 (34.4 - 36.4) | 72.0 |
| Oklahoma | 35.9 (34.8 - 37.0) | 74.0 |
| New Hampshire | 36.9 (35.0 - 38.8) | 76.0 |
| Texas | 37.3 (36.8 - 37.8) | 78.0 |
| Rhode Island | 37.6 (35.6 - 39.6) | 80.0 |
| Montana | 37.6 (35.5 - 39.7) | 82.0 |
| Michigan | 38.7 (38.0 - 39.4) | 84.0 |
| Wisconsin | 44.9 (43.9 - 45.9) | 86.0 |
| Washington | 45.0 (44.0 - 45.9) | 88.0 |
| South Dakota | 51.1 (48.4 - 53.9) | 90.0 |
| Oregon | 51.9 (50.6 - 53.1) | 92.0 |
| Vermont | 57.8 (54.5 - 61.1) | 94.0 |
| Idaho | 59.4 (57.1 - 61.7) | 96.0 |
| Wyoming | 63.0 (59.1 - 66.9) | 98.0 |
| North Dakota | 69.1 (65.6 - 72.5) | 100.0 |

**Supplementary Table 7:** AAMR per 100,000 for AMI with psychoactive substance use in adults aged 65 and above, stratified by State in the U.S., 1999-2020.
